# Plasma brain-derived neurotrophic factor concentrations are elevated in community-dwelling adults with sarcopenia

**DOI:** 10.1093/ageing/afaf024

**Published:** 2025-02-17

**Authors:** Jedd Pratt, Evgeniia Motanova, Marco V Narici, Colin Boreham, Giuseppe De Vito

**Affiliations:** Department of Sport and Exercise Sciences, Manchester Metropolitan University Institute of Sport, Manchester, M1 7EL, UK; Institute for Sport and Health, University College Dublin, Belfield, Dublin 4, Ireland; Department of Biomedical Sciences, CIR-Myo Myology Centre, Neuromuscular Physiology Laboratory, University of Padua, Padova, 35131, Veneto, Italy; Department of Biomedical Sciences, CIR-Myo Myology Centre, Neuromuscular Physiology Laboratory, University of Padua, Padova, 35131, Veneto, Italy; Institute for Sport and Health, University College Dublin, Belfield, Dublin 4, Ireland; Department of Biomedical Sciences, CIR-Myo Myology Centre, Neuromuscular Physiology Laboratory, University of Padua, Padova, 35131, Veneto, Italy

**Keywords:** sarcopenia, skeletal muscle health, screening, biomarkers, brain derived neurotrophic factor (BDNF), older people

## Abstract

**Background:**

The scalability of a blood-based sarcopenia assessment has generated interest in circulating markers that may enhance management strategies. Data regarding the relevance of brain derived neurotrophic factor (BDNF), a regulator of neuroplasticity, to sarcopenia in community-dwelling adults are scarce. We examined the association between plasma BDNF concentrations, sarcopenia and individual sarcopenia signatures in a well-characterised adult cohort.

**Methods:**

Participants included 246 men and women aged 50–82 years (mean age = 63.6 years; 52% female). Muscle strength and skeletal muscle index (SMI) were assessed by hand dynamometry and dual-energy X-ray absorptiometry. Plasma BDNF concentrations were determined, in duplicate, with commercially available enzyme-linked immunosorbent assays. Sarcopenia and individual signatures of sarcopenia (i.e*.* low grip strength or low SMI) were diagnosed according to the EWGSOP2 algorithm.

**Results:**

Plasma BDNF concentrations were 47.6% higher in participants with sarcopenia than controls (*P* = 0.005), and demonstrated acceptable diagnostic accuracy (areas under the curves = 0.702, 95%CI = 0.597–0.806, *P* = 0.002, optimal cut-off >1645 pg/ml). Plasma BDNF concentration >1645 pg/ml was associated with 2.83 greater odds for sarcopenia (95%CI = 1.13–7.11, *P* = 0.027), than ≤1645 pg/ml, whilst a BDNF Z-score ≥2 was associated with 5.14 higher odds for sarcopenia (95%CI = 1.16–22.82, *P* = 0.031), than a Z-score <1. Covariates included sex, age, body mass index, habitual physical activity, smoking status, alcohol consumption, comorbidity and educational attainment.

**Conclusion:**

Circulating BDNF concentrations are elevated in community-dwelling men and women with sarcopenia, which may reflect increased neuromuscular remodelling in these people. Our findings complement existing data, supporting the presence of an intricate relationship between neural integrity and skeletal muscle health. Future studies are needed to establish the mechanistic pathways that may underpin the associations.

## Key Points

- Plasma brain derived neurotrophic factor concentrations were significantly elevated in community dwelling adults with sarcopenia.
- Plasma brain derived neurotrophic factor concentrations were associated with up to ~5 times higher odds for sarcopenia, depending on brain derived neurotrophic factor index.
- Future studies are needed to elucidate underpinning mechanistic pathways.

## Introduction

The progressive deterioration of skeletal muscle mass and function that accompanies ageing imposes a heavy burden on patients and health systems globally. Besides considerable healthcare costs [1], presenting with low skeletal muscle function and mass, officially termed sarcopenia, increases the risk of a multitude of negative health outcomes, such as cognitive and mobility impairment [2], loss of independence [3] and hospitalisation [4]. Given that sarcopenia is highly prevalent, affecting ⁓40% of people aged over 80 years [5], there is an urgent need to identify and develop effective management strategies.

Barriers associated with attaining relevant muscle mass and function data, including the cost, availability and precision of equipment and inaccuracies due to comorbidities or injury, have impeded the clinical management of the disease [6]. Increasing attention has been given to the potential utility of a blood-based assessment, which may help alleviate existing barriers, and enhance strategies seeking to combat sarcopenia. Accumulating evidence supports the use of circulating biomarkers for providing insight into the health of skeletal muscle, with existing biomarkers selected according to their relevance to physiological pathways governing skeletal muscle regulation [7–9]. Although the factors underlying sarcopenia are multifactorial [10–13], increasing data suggest neuromuscular processes are central mediators. Several studies have demonstrated circulating markers of neural and neuromuscular health [e.g. neurofilament light chain (NfL)—a marker of axonal damage; and C-terminal agrin fragment (CAF)—a marker of neuromuscular junction stability] to be associated with sarcopenia and individual signatures of sarcopenia (i.e*.* low muscle strength or low muscle mass) [7, 8, 14]. Although CAF and NfL are amongst the most promising candidate biomarkers of skeletal muscle health, considering that large heterogeneity exists in CAF concentrations across adulthood [15] and accurately determining NfL concentrations can be costly, their suitability as biomarkers in the context of ageing requires further consideration.

Another physiologically plausible biomarker is brain-derived neurotrophic factor (BDNF), a member of a broader group of neurotrophins that are potent regulators of neuronal growth and plasticity [16]. BDNF is an important contributor to neuromuscular junction stability, a critical factor underpinning skeletal muscle integrity [12, 17]. BDNF further supports skeletal muscle regeneration and repair through its involvement in regulating satellite cell proliferation and differentiation [18, 19], and promoting insulin sensitivity [20]. Importantly, circulating BDNF levels can be easily determined from peripheral blood samples using commercially available enzyme-linked immunosorbent assays (ELISAs) [21]. Perhaps surprisingly therefore, a comprehensive evaluation of the relevance of circulating BDNF to sarcopenia in community-dwelling adults is yet to be performed. Although studies have reported associations in haemodialysis patients [22], and patients suffering from heart failure [23], chronic obstructive pulmonary disease [24] and Parkinson’s disease [25], only one has included community-dwelling adults (sample size n = 71, no males included) [9]. A further study examined the association between circulating BDNF and frailty [26], although these people presented with considerable physiological and neurological decline, and so, the findings may not be informative to otherwise healthy community-dwelling adults. Existing findings are inconsistent, and the overall quality of evidence is affected by small sample sizes (n = 20–71) [9, 22, 23] and lack of consideration for health and lifestyle factors, such as habitual physical activity, alcohol consumption, smoking status and sleep quality, that may confound the relationship between circulating BDNF and skeletal muscle traits [9, 22–25, 27]. There remains a need to determine the relevance of circulating BDNF levels to skeletal muscle health in community-dwelling men and women, with appropriate consideration for confounding factors.

Herein, this study aimed to determine the relevance of plasma BDNF concentrations to sarcopenia and individual signatures of sarcopenia (i.e*.* low muscle strength or low muscle mass) in a comprehensively characterised cohort of community-dwelling middle-aged and older men and women.

## Methods

### Study sample

Participants were recruited via the GenoFit study, an analysis of genetic, health and lifestyle traits of people living in Ireland between September 2017 and October 2020 [28]. Participation involved a single assessment lasting 1 h, where a blood sample was drawn, and a range of phenotypic and lifestyle data were collected. For the present study, all participants aged ≥50 years (n = 246) were selected from the overall pool of GenoFit participants with plasma BDNF concentration data (n = 720). We focused on people ≥50 years of age as a distinguishable decline in skeletal muscle health has been observed from this point in this cohort [29]. To be eligible, participants had to have no musculoskeletal injury or neurological pathology that may affect muscle strength and/or mass (e.g*.* injury to the hand, wrist or arm, peripheral neuropathies such as carpal tunnel syndrome, or muscular dystrophies) and be able to provide informed consent. Ethical approval was granted by the relevant Institutional Ethics Committee and all participants gave written informed consent.

### Body composition and muscle strength analysis

**Figure 1:**
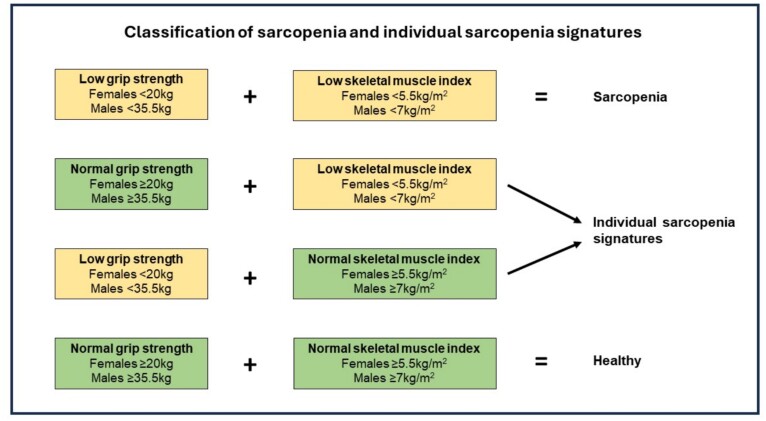
Classification of sarcopenia and individual sarcopenia signatures.

Body mass and height were assessed using a SECA (SECA, Hamburg, Germany) weighing scales and stadiometer, and a body mass index (BMI) was calculated as body mass divided by height squared (kg/m^2^). Dual-energy X-ray absorptiometry (DXA) (Lunar Prodigy, GE Healthcare Technologies, USA) determined body composition of participants in a rested state, and a skeletal muscle index (SMI) was calculated as appendicular lean mass (combined lean mass of limbs) divided by height squared (kg/m^2^). Muscle strength was assessed using a handheld dynamometer (JLW Instruments, Chicago, IL, USA) according to a previously described protocol [29]. The average of the highest score from each hand was used in the analysis [30]. Sarcopenia and individual sarcopenia signatures (i.e*.* low SMI or low grip strength) were classified using the EWGSOP2 algorithm incorporating cut-points recently suggested by Westbury and colleagues [31, 32]. Sarcopenia was diagnosed when low SMI and low grip strength were present, using the following thresholds: low grip strength <35.5 kg for men and <20 kg for women; low SMI <7 kg/m^2^ for men and <5.5 kg/m^2^ for women. A flowchart for the classification of sarcopenia and individual sarcopenia signatures is presented in Figure 1.

### Blood sampling and plasma brain-derived neurotrophic factor measurement

Blood samples were collected from participants in a rested state, from the medial cubital vein using ethylenediaminetetraacetic acid vacutainers (BD Vacutainer) and processed as described before [33]. Plasma BDNF concentrations were measured, in duplicate, using commercially available ELISAs (#ab212166, Abcam, Cambridge, UK) according to manufacturer’s guidelines. Briefly, 50 μL of diluted sample (20-fold dilution) or standard was added to each well, followed by 50 μL of BDNF antibody. The plate was incubated at room temperature for 1 h on a plate shaker at 400 rpm. Each well was washed three times, and 100 μL of TMB development solution was added to each well prior to further incubation for 10 min. Lastly, 100 μL of stop solution was added to each well and the measurements were recorded at 450 nm (Infinite F50 Plus, Tecan, Männedorf, Switzerland). Concentrations of BDNF were normalised against internal calibrator concentration and established by standard curve interpolation and correction for dilution factor. The inter-assay coefficient of variation was below 11% and the intra-assay coefficient of variation was below 4%.

### Covariates

Habitual physical activity, smoking status, alcohol consumption, educational attainment and comorbidity were assessed using self-reported questions as described previously [29] (detailed in Appendix 1 Supplementary Material).

### Statistical analyses

Independent-sample Student’s t-test and Chi-square tests determined differences in participant characteristics, whilst Pearson’s correlation coefficient examined the relationship between plasma BDNF and age. Data normality was examined using residual plots, and skewness and kurtosis data. Multiple linear regression analyses were used to assess the relationship between plasma BDNF level, grip strength and SMI, whilst analyses of covariance and multiple logistic regression models were used to determine the relationship between plasma BDNF concentration, sarcopenia and individual sarcopenia components (low grip strength or low SMI). Models were adjusted for sex, age, BMI, habitual physical activity, educational attainment, smoking status, alcohol intake and comorbidity. Receiver operating characteristic (ROC) analyses were used to examine the diagnostic value of plasma BDNF for sarcopenia and individual sarcopenia components, using Youden’s index to determine optimal cut-points. Areas under the curves (AUCs) were interpreted as: 0.5–0.69 = poor, 0.7–0.79 acceptable, 0.8–0.89 = good and 0.9–1.0 excellent. To establish the value of circulating BDNF in identifying sarcopenia we examined odds for sarcopenia using several plasma BDNF indices, including cut-point established from ROC analysis, and plasma BDNF Z-scores, either categorised as <1, 1–1.99 or ≥2, or coded as a continuous variable. Z-scores were calculated as the difference between the BDNF concentration of an individual and the mean BDNF concentration from a young adult population (18–39 year olds from this study sample), divided by the SD of the young adult population (young adult mean = 1297.0 pg/ml and SD = 1016.0 pg/ml). We used SPSS (Version 28, IBM SPSS Inc., Chicago, IL, USA) for statistical analyses and GraphPad Prism (Version 9.3.1, Prism, San Diego, CA, USA) for visualisations. Statistical significance was set at *P* < 0.05.

## Results

Participant characteristics according to sex are detailed in Table 1. In total, 246 adults aged between 50 and 82 years were included in this study (females: n = 129, mean age = 63.4 years; males: n = 117, mean age = 63.8 years). Plasma BDNF concentrations did not differ significantly between men and women (*P* = 0.414).

**Table 1:** Participant characteristics.

Parameter	Total (n = 246)	Males (n = 117)	Females (n = 129)	*P*-value
Age (years)	63.6 (7.7)	63.8 (8.1)	63.4 (7.4)	0.709
Age range (years)	50–82	50–82	50–80	
Height (cm)	168.7 (9.4)	175.8 (6.8)	162.3 (6.4)	<0.001
Body mass (kg)	72.7 (14.8)	82.0 (12.8)	64.2 (11.1)	<0.001
BMI (kg/m^2^)	25.4 (3.6)	26.5 (3.3)	24.3 (3.7)	<0.001
Skeletal muscle index (kg/m^2^)	7.3 (1.4)	8.4 (1.0)	6.4 (0.9)	<0.001
Grip strength (kg)	36.3 (11.3)	45.4 (8.5)	28.2 (6.0)	<0.001
Low grip strength	26 (10.6)	10 (8.5)	16 (12.4)	
Low skeletal muscle index	24 (9.8)	10 (8.5)	14 (10.9)	
Sarcopenia	23 (9.3)	12 (10.3)	11 (8.5)	
Plasma BDNF level (pg/ml)	1520.6 (920.3)	1470.1 (820.2)	1566.3 (1003.4)	0.414
Habitual physical activity^a^	3.8 (2.3)	3.9 (2.4)	3.8 (2.3)	0.764
Alcohol consumption (units/week)	8.6 (6.9)	10.9 (7.6)	6.5 (5.6)	<0.001
*Education, n (%)*				
None or primary education	6 (2.4)	2 (1.7)	4 (3.1)	0.115
Lower secondary	24 (9.8)	12 (10.3)	12 (9.3)	
Higher secondary	43 (17.5)	22 (18.8)	21 (16.3)	
Third-level degree	127 (51.6)	52 (44.4)	75 (58.1)	
Postgraduate degree	46 (18.7)	29 (24.8)	17 (13.2)	
*No. of diseases/disorder, n (%)*				
None	70 (28.5)	34 (29.1)	36 (27.9)	0.891
One	67 (27.2)	33 (28.2)	34 (26.4)	
Two or more	109 (44.3)	50(42.7)	59 (45.7)	
*Smoking status, n (%)*				
Never smoked (<100 cigarettes)	126 (51.3)	54 (46.2)	72 (55.8)	0.261
Previously smoked (>100 cigarettes)	52 (21.1)	29 (24.7)	23 (17.8)	
Currently smoke (>100 cigarettes)	68 (27.6)	34 (29.1)	34 (26.4)	

^a^Days per week performing at least 30 min of moderate intensity exercise; BMI = body mass index; BDNF = brain derived neurotrophic factor.

### Plasma BDNF concentration, sex, age and skeletal muscle health

In women, a significant but weak positive correlation was observed between plasma BDNF concentration and age (*P* = 0.026) (Figure S1, Appendix 2 Supplementary Material). No such association was observed in men (*P* = 0.877), or in the collective sample (*P* = 0.141). Multiple linear regression revealed plasma BDNF concentration to be negatively associated with grip strength and SMI (both *P* < 0.001), after adjustment for sex, age, BMI, comorbidity, educational attainment, habitual physical activity, alcohol consumption and smoking status (Table S1, Appendix 3 Supplementary Material).

### Plasma BDNF concentrations, sarcopenia and individual sarcopenia signatures

**Figure 2:**
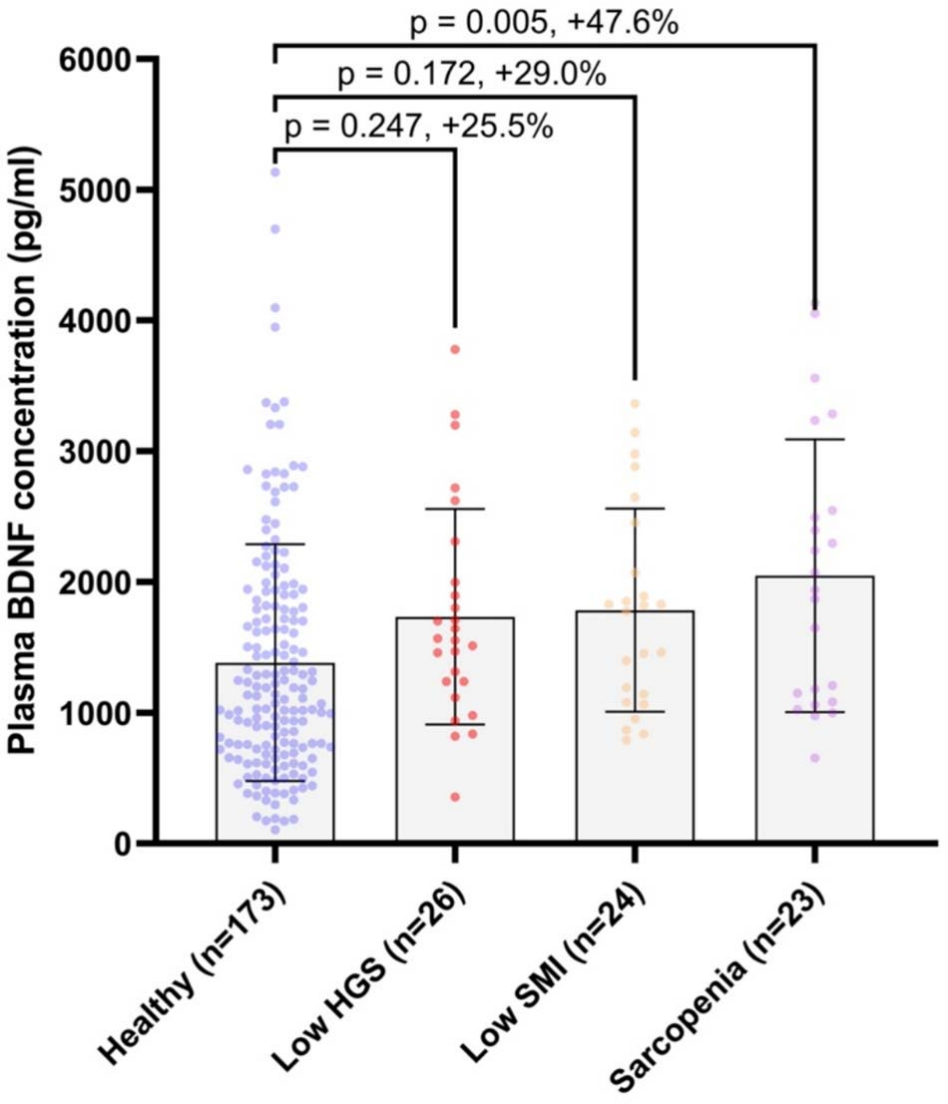
Plasma BDNF concentrations according to sarcopenia status in people ≥50 years of age. HGS = hand grip strength; SMI = skeletal muscle index; error bars = mean ± SD.

Plasma BDNF concentrations were 47.6% higher in those with sarcopenia (p = 0.005), compared to healthy controls (Figure 2). These associations were robust to adjustment for sex, age, BMI, habitual physical activity, smoking status, alcohol consumption, comorbidity and educational attainment (Table S2, Appendix 4 Supplementary Material). Plasma BDNF concentrations were also 29.0% higher in people with low SMI only, and 25.5% higher in people with low grip strength only, compared to healthy controls, although these differences did not reach statistical significance before, or after, covariate adjustment.

**Figure 3:**
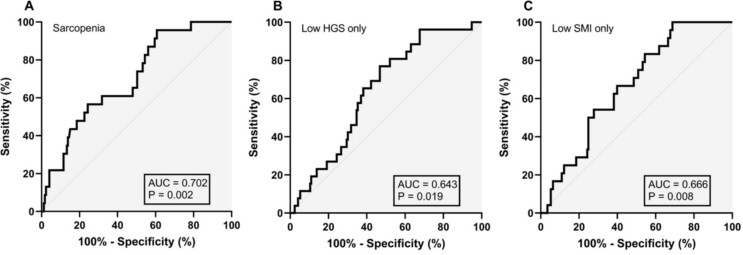
Diagnostic value of plasma brain derived neurotrophic factor concentration for sarcopenia (A), low HGS only (B), and low SMI only (C).

Plasma BDNF concentrations demonstrated acceptable diagnostic value for sarcopenia with an AUC of 0.702 (95%CI = 0.597–0.806, *P* = 0.002), and corresponding optimal cut-point of >1645 pg/ml (60.9% sensitivity and 68.2% specificity) (Figure 3). The AUCs for low grip strength only and low SMI only were 0.643 (95%CI = 0.544–0.741, *P* = 0.019) and 0.666 (95%CI = 0.569–0.763, *P* = 0.008) with optimal diagnostic thresholds of >1237 pg/ml (76.9% sensitivity and 53.2% specificity) and > 1396 pg/ml (66.7% sensitivity and 60.1% specificity).

People with plasma BDNF >1645 pg/ml (optimal cut-off from ROC analysis) had 2.83 greater odds for sarcopenia (95%CI = 1.13–7.11, *P* = 0.027), than those with concentrations ≤1645 pg/ml, after adjustment for a panel of relevant covariates (Table 2). Stratification by plasma BDNF Z-score (categorised as <1, 1–1.99 and ≥ 2), revealed that people with a Z-score between 1 and 1.99 had 3.18 greater odds for sarcopenia (95%CI = 1.04–9.72, *P* = 0.043), and people with a Z-score ≥2 had 7.31 greater odds for sarcopenia (95%CI = 2.18–24.55, *P* = 0.001), than people with a Z-score < 1 (Table 2). Following adjustment for the same panel of covariates, significantly higher odds for sarcopenia remained for people with a Z-score ≥ 2 (OR = 5.14, 95%CI = 1.16–22.82, *P* = 0.031), but not for people with a Z-score between 1 and 1.99 (*P* = 0.320). Considering plasma BDNF Z-score as a continuous variable, a 1 unit increase in Z-score was associated with 1.73 greater odds for sarcopenia (95%CI = 1.09–2.75, *P* = 0.020), in the fully adjusted model (Table 2).

**Table 2:** Odds for sarcopenia according to plasma BDNF indices in people aged ≥50 years.

	Model 1		Model 2		Model 3	
BDNF index	OR (95% CI)	*P*-value	OR (95% CI)	*P*-value	OR (95% CI)	*P*-value
>1645 pg/ml^a^	2.84 (1.18–6.84)	0.020	2.76 (1.14–6.69)	0.025	2.83 (1.13–7.11)	0.027
Z-score 1–1.99^b^	3.18 (1.04–9.72)	0.043	2.14 (0.66–6.98)	0.206	1.94 (0.53–7.10)	0.320
Z-score ≥ 2^b^	7.31 (2.18–24.55)	0.001	5.25 (1.39–19.84)	0.015	5.14 (1.16–22.82)	0.031
Z-score^c^	1.80 (1.19–2.72)	0.005	1.70 (1.10–2.62)	0.017	1.73 (1.09–2.75)	0.020

^a^Optimal cut-point from receiver operating characteristic analysis.

^b^Referenced against Z-score < 1.

^c^Per 1 unit increase in Z-score coded as a continuous variable.

Model 1 = unadjusted; Model 2 = adjusted for sex, age, body mass index; Model 3 = model 2 and habitual physical activity, smoking status, alcohol consumption, educational attainment, comorbidity.

## Discussion

Our principal findings show that: i) plasma BDNF concentrations are negatively associated with grip strength and SMI; ii) plasma BDNF concentrations are ~48% higher in those with sarcopenia, compared to healthy controls; iii) plasma BDNF is associated with up to ~5 times greater odds for sarcopenia, depending on BDNF index and has acceptable diagnostic accuracy for sarcopenia, although larger studies with a higher representation of sarcopenic people are needed to further elucidate the diagnostic value of BDNF.

This is the first study to demonstrate that plasma BDNF concentrations are significantly elevated in community-dwelling men and women with sarcopenia. Encouragingly, circulating BDNF appears to be progressively associated with skeletal muscle degradation, such that healthy people have the lowest concentrations, followed by people with individual sarcopenia signatures (low grip strength or low SMI), followed by people with sarcopenia. This incremental association is suggestive of a causal relationship although we recognise the cross-sectional design of this study precludes a definitive inference to be made. Moreover, despite large differences in plasma BDNF of 25.5% and 29% between healthy controls and people with either low grip strength or low SMI, statistical significance was not reached, and so further examination of the potential presence of an incremental association between circulating BDNF and stages of skeletal muscle decline is required. Future studies including participants that span the older adulthood period may be particularly informative against whether age mediates the strength of the association between circulating BDNF and skeletal muscle health. Nevertheless, our findings do suggest that plasma BDNF may have clinical value in identifying people who may be vulnerable to poor skeletal muscle outcomes for inclusion in therapeutic interventions. Indeed, using several indices, including Z-score categories and the optimal cut-off derived from ROC analyses, higher plasma BDNF concentrations were associated with substantially higher odds for sarcopenia, after adjustment for relevant covariates.

Existing studies pertaining to circulating BDNF and skeletal muscle health have predominantly investigated diseased populations [22–25], and in contrast to our findings, have observed lower concentrations of BDNF in sarcopenic people compared to healthy controls. Another study observed lower concentrations of plasma BDNF in frail older adults compared to non-frail older adults [26]. It is noteworthy however, that the poorer cognitive status of the participants of the above studies [22–26] compared with the healthy older adults included in the present study, is a likely contributor to these conflicting findings. For example, BDNF is known to increase in early stages of Alzheimer’s disease, as a protective mechanism against early neurodegeneration, but then decreases in more severe, chronic cases of neural deterioration [34]. With this in mind, the significant neurodegeneration present in participants of the aforementioned studies [22–26] may explain the lower BDNF concentrations observed in states of sarcopenia where further neurodegeneration is likely to be present. Only one study has included healthy community-dwelling older adults. However, this study was relatively small (n = 71) and included females only [9]. Nevertheless, our findings align with those of da Costa Teixeira *et al.* [9], and provide supportive evidence of substantially elevated concentrations of plasma BDNF in community-dwelling adults with sarcopenia. In light of the available evidence, we propose that in otherwise healthy community-dwelling middle-aged and older adults, the higher concentrations of BDNF in sarcopenia may reflect a greater level of neuromuscular remodelling present in these individuals. This aligns with accumulating data indicating skeletal muscle health to be powerfully mediated by neural and neuromuscular factors, particularly in older adulthood [7, 8, 35, 36]. However, it is important to note that although this is a plausible hypothesis that may help to explain our findings, future mechanistic studies are ultimately needed to confirm or refute this. It is also noteworthy, that the overall body of literature relating to the relationship between circulating BDNF and skeletal muscle traits in healthy adults is scarce and incongruent. Our current understanding will benefit greatly from future large-scale studies, incorporating well-defined cohorts, accurate determination methods and appropriate adjustment for important covariates.

In this regard, we propose several important targets for future research, the first of which is a longitudinal assessment of BDNF trajectories across adulthood. The cross-sectional design of the present study, coupled with the mean age of the cohort being ~64 years, limits our interpretation of how BDNF concentrations may change across older adulthood. Several studies have shown negative, although modest associations between age and BDNF concentration [37, 38], whilst others have shown positive associations [39]. In the present study we found no association between age and BDNF concentrations across the full sample, whilst after stratification by sex, a weak positive correlation was shown in females, and no correlation was shown in males. Although the precise mechanisms underpinning the variability in the reported associations between age and BDNF remain to be determined, the age distribution of the cohorts coupled with differences in physiological and cognitive health status may help to explain the conflicting findings. Indeed, given that BDNF concentrations typically increase in early stages of neural disruption and later decline in the presence of more significant neurodegeneration [34], it is plausible, that the different age-related trajectories of BDNF may be at least partly due to differences in the health status of participants across studies. Nevertheless, there remains is a strong need for a longitudinal assessment of circulating BDNF within an age-diverse sample of community-dwelling adults, to contextualise our findings and provide clarity on typical age-related trajectories of BDNF. Secondly, physical activity is a known modulator of circulating BDNF concentrations. Although weekly engagement in physical activity was adjusted for in the present study, there is a need for a more precise characterisation of physical activity, examining the effect of duration, type, and intensity, on the associations between habitual BDNF concentrations and skeletal muscle. Although it is well-established that acute physical activity transiently increases BDNF concentrations, the chronic effects of physical activity variations are less clear [40]. With this in mind and considering that physical activity is an important factor governing skeletal muscle health, it is plausible that there may be an interaction between engagement in different activity types and intensities, and the associations between BDNF and sarcopenia.

Several strengths and weakness to our work should be acknowledged. The strengths include: i) the well characterised cohort including an equal proportion of men and women; ii) comprehensive phenotyping that facilitated appropriate adjustment for important covariates, ultimately adding to the robustness of our findings. The limitations include: i) the cross-sectional design precludes the establishment of causality and statistical power was not calculated as a convenience cohort sample was used; ii) although conflicting, some data suggest circulating BDNF concentrations differ depending on time of day [41], and so, recording time of blood sample collection may have been a valuable addition to this study; iii) we only recruited community-dwelling adults living in Ireland, and so, our findings may not be transferable to other populations. More studies are needed, therefore, to contextualise our findings and determine the relevance of circulating BDNF concentrations to skeletal muscle health in other populations; iv) we did not assess cognitive status of participants, which may have provided support to our hypothesis that the increase in BDNF concentration represents a compensatory mechanism for early neurodegenerative processes, in otherwise healthy community-dwelling adults. However, no participants self-reported having cognitive impairment in the medical history questionnaire; v) including an assessment of gait speed, would have provided further insight into the relevance of plasma BDNF levels to physical function; vi) given that some data suggest that plasma BDNF concentrations vary according to menstrual cycle phase and status [42], and our cohort included women aged from 50 years, controlling for menstrual status may have facilitated a clearer examination of the relationship between BDNF and skeletal muscle health in women.

To conclude, our work demonstrates for the first time, that plasma BDNF concentrations are substantially elevated in middle-aged and older community-dwelling men and women with sarcopenia. The increased concentration of BDNF in sarcopenic people may be the product of a compensatory response to heightened neuromuscular remodelling. It is noteworthy however, that this is an under-researched area, and more studies are needed to contextualise our findings and provide mechanistic insight into the observed associations.

## Supplementary Material

aa-24-1062-File005_afaf024

## Data Availability

Data may be made available upon reasonable request to the corresponding author.
