# Supplementary material for: Plasma brain-derived neurotrophic factor concentrations are elevated in community-dwelling adults with sarcopenia
Source: Age Ageing. 2025 Feb 17;54(2):afaf024. doi: 10.1093/ageing/afaf024 (PMC11831035; doi:10.1093/ageing/afaf024)
Supplement: aa-24-1062-File005_afaf024 [file aa-24-1062-file005_afaf024.docx]

**SUPPLEMENTARY MATERIAL**

**Plasma brain-derived neurotrophic factor concentrations are elevated in community-dwelling adults with sarcopenia**

**Contents:**

APPENDIX 1 Details of self-reported questions used to assess habitual physical activity, smoking status, alcohol consumption, educational attainment and comorbidity.

APPENDIX 2 Figure S1. Association between plasma brain derived neurotrophic factor (BDNF) concentration and age in the full sample (A), in females (B) and in males (C)

APPENDIX 3 Table S1. Adjusted associations between plasma brain-derived neurotrophic factor (BDNF) concentrations, grip strength and skeletal muscle index

APPENDIX 4 Table S2. Adjusted associations between plasma brain derived neurotrophic factor (BDNF) level and sarcopenia status

APPENDIX 1

**Details of self-reported questions used to assess habitual physical activity, smoking status, alcohol consumption, educational attainment and comorbidity:**

“How many days per week do you do at least 30 min of moderate-intensity exercise that increases your breathing and heart rate (e.g. brisk walking, jogging, cycling, swimming)?”

“Smoking categorised as (1) never smoked (never smoked/smoked <100 cigarettes in lifetime), (2) previous smoker (smoked ≥100 cigarettes in lifetime but no longer smoking), and (3) current smoker (smoked ≥100 cigarettes in lifetime and currently smoking)”;

“On average, how many standard drinks do you drink per week?"

"What is the highest level of education you have completed to date (no formal education, primary, lower secondary, higher secondary, third level, or postgraduate)?"

"Have you ever received a medical diagnosis from a doctor for any of the following conditions?"

*The total number of diseases and/or disorders was calculated for every participant.*

- **Cancer**: blood leukaemia, breast, colon, kidney, lung, ovarian, pancreas, prostate, skin
- **Heart disease/disorder:** abnormal heart beat arrhythmia, angina, heart attack, high blood pressure, high cholesterol, stroke
- **Skin disorder**: eczema, melasma, psoriasis, rosacea, scleroderma
- **Digestive/bowel disorder**: coeliac disease, Crohn’s disease, fatty liver disease, inflammatory bowel disease, irritable bowel syndrome, ulcerative colitis
- **Breathing disorder**: asthma, chronic obstructive pulmonary disease, emphysema, cystic fibrosis, idiopathic pulmonary fibrosis, lung fibrosis, sarcoidosis
- **Bone/joint disorder**: ankylosing spondylitis spondylarthritis, enteropathic arthritis, osteoarthritis, psoriatic arthritis, reactive arthritis, rheumatoid arthritis, osteoporosis
- **Pain disorder**: chronic back pain, migraine
- **Mental health condition**: anxiety, bipolar disorder, depression, schizophrenia
- **Neurological**: Alzheimer’s, age related macular degeneration, autism, epilepsy, motor neurone disease, multiple sclerosis, Parkinson’s
- **Diabetes**: gestational diabetes, type 1 diabetes, type 2 diabetes
- **Other**: alopecia, endometriosis, haemochromatosis, kidney disease, kidney failure, Lupus, Sjogren Syndrome, thyroid disorder, other (please specify)

APPENDIX 2


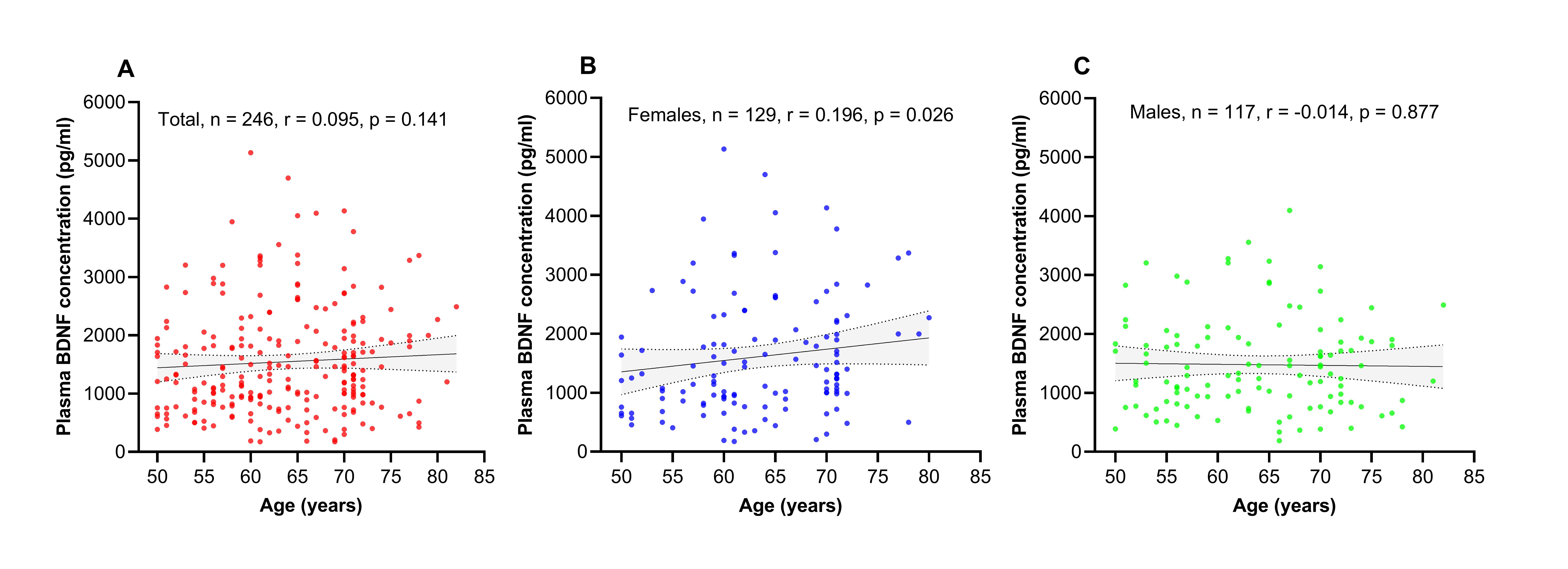


**Figure S1.** Association between plasma brain derived neurotrophic factor (BDNF) concentration and age in the full sample (A), in females (B) and in males (C)

APPENDIX 3

| **Table S1.** Adjusted associations between plasma brain-derived neurotrophic factor (BDNF) concentrations, grip strength and skeletal muscle index | | | | |
| --- | --- | --- | --- | --- |
|  | Plasma BDNF ^b^ | | | |
| Dependent variable ^a^ | β | 95% CI | p-value | R^2^ |
|  |  |  |  |  |
| Grip strength | -1.824 | -2.818 - -0.830 | <0.001 | 0.640 |
| Skeletal muscle index | -0.264 | -0.391 - -0.137 | <0.001 | 0.571 |
| ^a^ = Adjusted for sex, age, body mass index, comorbidity, habitual physical activity, smoking status, educational attainment and alcohol consumption; ^b^ = per 1 SD increase in BDNF concentration | | | | |

APPENDIX 4

| **Table S2.** Adjusted associations between plasma brain derived neurotrophic factor (BDNF) level and sarcopenia status | | | | | |
| --- | --- | --- | --- | --- | --- |
| **Model** | | **Healthy (n=343)** | **Sarcopenia (n=49)** | **Low SMI (n=63)** | **Low HGS (n=60)** |
|  | | *Plasma BDNF (pg/ml)* | | | |
| Model 1 | 1426.3 (54.1) | | 1912.8 (173.8)* | 1623.7 (173.5) | 1651.67 (165.9) |
| Model 2 | 1434.9 (54.3) | | 1829.6 (181.0)* | 1678.9 (174.9) | 1676.8 (165.5) |
| Data displayed as mean (standard error of mean); Model 1 = adjusted for sex, age and body mass index; Model 2 = Model 1 and habitual physical activity, alcohol consumption, smoking status, educational attainment and comorbidity; SMI = skeletal muscle index; HGS = hand grip strength * p<0.05 | | | | | |
